# Leadership and gender perspective in hospital physiotherapy units

**DOI:** 10.1038/s41598-024-60820-1

**Published:** 2024-05-01

**Authors:** Mercedes Ferrando Margelí, Aitor Garay Sánchez, Elena Andrade-Gómez, Carmen Suarez-Serrano, Yolanda Marcén-Román

**Affiliations:** 1 Miguel Servet University Hospital, Zaragoza, Spain; 2 GIIS086 Group, Instituto Investigación Sanitaria Aragón, Zaragoza, Spain; 3 Department of Anatomy and Human Histology, Faculty of Medicine, University of Zaragoza, Zaragoza, Spain; 4 Department of Nursing, Faculty of Health Sciences, University of La Rioja, C/Duquesa de la Victoria, nº 88, 26004 Logroño, La Rioja Spain; 5 Department of Physiotherapy, Faculty of Nursing, Physiotherapy and Podiatry, University of Seville, Seville, Spain

**Keywords:** Gender, Communication, Leadership, Health Professions, Physiotherapy, Health care, Risk factors

## Abstract

Analyze the gender stereotypes present in the leaders of the Hospital Physiotherapy Units, determine the level of acceptance of female leadership and identify which factors influence these perceptions. Observational, descriptive, exploratory and cross-sectional study. The study subjects are the census of leaders of the Physiotherapy Units of public hospitals. The measurement instruments used are the Acceptance of Female Leadership Questionnaire (ACT-LM), and the sociodemographic and job-related variables. Most of the leaders of the hospital physiotherapy units were women (69.4%) physiotherapists. Gender stereotypes emerge in the dimension of Instrumental Characteristics, with respondents not fully agreeing that women were sufficiently competitive (18.7%) or ambitious (20.8%) to be successful in the world of work. These data were influenced by gender, showing that men have a higher regard for female leadership abilities than women themselves. In the dimension of Acceptance of Female Leadership, 17.4% of those surveyed did not fully agree that women can rise to the same extent as men. Most of the leaders of the physiotherapy units in public hospitals in Spain are women, this is reversed in favor of men in highly complex hospitals. The stereotype persists, especially among women, that they do not have enough ambition and competitiveness to succeed in the world of work.

## Introduction

Gender is a fundamental aspect that has been widely studied from different perspectives and in different areas, including management and leadership^1^. Gender stereotypes refer to generalized beliefs and expectations about characteristics, abilities, interests, and behaviors that are assumed to be appropriate for men and women in a certain society^2^.

In recent decades, there has been an increase in the participation of women in all areas of work. However, inequality persists in the degree of occupation of management positions^3,4^. The relationship between leadership and gender has been evolving, from “The Wall”, which referred to men working and women at home, to the “Glass Ceiling”, which refers to invisible barriers, linked to gender inequalities rooted in society, which hinder the professional progression of women^5^. Subsequently, “The Labyrinth”^6^ emerges, which refers to the greatest number of obstacles that women must overcome to access leadership positions until the “Glass Cliff” that warns that women access positions in which there is greater risk of failure^7^.

In health professions, gender continues to be a determining factor that is reflected in the professional trajectories of men and women^8^. Gender stereotypes permeate not only professionals, but also patients, perpetuating themselves in the organizational culture of the healthcare field^9^. The low presence of women in management and decision-making positions can lead to a lack of understanding of women’s working conditions and the specific needs of women in terms of health care. This can lead to these issues being neglected in health policies, thus compromising gender equality in the exercise of the right to health^9^.

In today's society, gender stereotypes remain entrenched, occupying a significant space both in the media and in the field of research. In the healthcare field, recent studies have indicated that gender stereotypes continue to be an obstacle for women leaders^10^, who must face challenges to demonstrate their competence and leadership skills. In the field of physiotherapy, there is hardly any national or international research that specifically addresses gender stereotypes in leadership^11^.

In nursing, an eminently female profession, the influence of gender on leadership has been widely studied^12^, concluding that gender stereotypes continue to be a barrier to the development of nurses’ leadership. In this profession, the influence that aspects such as age, lack of training and work environment have on the possibilities of leadership development in women has been studied^12^.

According to 2022 data from the National Institute of Statistics^13^, physiotherapy is also a predominantly female profession, 61.64% of women compared to 38.35% of men, it is important to analyze the current situation of leadership in physiotherapy in the healthcare field to identify and apply areas of improvement that promote gender equity.

The inclusion of the gender perspective in research allow us to visualize the political and institutional influences that contribute to the persistence of these inequalities^14^. However, to date, in the field of physiotherapy, research on leadership^11^ and gender^15^ is scarce and little explored.

The main objective of this study is to analyze the gender stereotypes present in the leaders of Hospital Physiotherapy Units, as well as to determine their level of acceptance of female leadership. Likewise, it seeks to identify the factors that influence these perceptions, to contribute to progress towards more inclusive and equitable leadership in the field of physiotherapy.

## Methods

### Study design

A study has been carried out with quantitative methodology, through an observational, descriptive, and cross-sectional design.

### Participants

The study population was health professionals who performed management and leadership roles in the Physiotherapy Units of the General Hospital network of the Spanish public Health System. Given that this is a relatively small population, it was considered that the census would constitute the study population.

Inclusion criteria:

- Heads of the Physiotherapy Units of the General Hospitals of the Public Health System.

The exclusion criteria included those heads or managers of the Physiotherapy Units in:,, private hospitals, privately managed public hospitals, military and NGO hospitals, specialized public hospitals, medium and long-stay public hospitals, and mental health hospitals.

### Procedure

Prior to carrying out this research, the Clinical Research Ethics Committee of Aragon (CEICA) Favorable Report was obtained, file number PI21/009, and after the consent from the author of the ACT-ML questionnaire^16^ for its use, was requested and also obtained.

Contact with the study population was made by requesting participation from the entire population through the representatives of each region, members of the Quality Group of the General Council of Colleges of Physiotherapists of Spain. To obtain a higher response rate, telephone contact was established with the study subjects who had not been previously contacted, requesting participation. For this, the telephone numbers registered in the National Hospital Catalogue^17^ were used.

Data collection was carried out using a questionnaire designed through Google Forms that included the ACT-ML questionnaire, sociodemographic and job-related questions. In the introduction prior to the questionnaire, the objective of the study and the privacy policy of the digital platform were described. Likewise, participants were informed that their acceptance to participate gave consent to the researchers to use the data extracted from the questionnaire. The design of the questionnaire was carried out in such a way that the extracted data matrix maintained the coding of the responses.

The data were collected during the period between June and October 2022. Once this period ended, the data was transferred from the Excel document provided by Google Forms to the SPSS statistical package (Statistical Package for the Social Sciences), version 28 for analysis.

### Measure instrument

The measurement tool selected to carry out this study was the questionnaire designed by Dr. María Laura Lupano Perugini^16^, “Acceptance of Female Leadership” (ACT-ML), developed to evaluate unfavorable attitudes towards women leaders. This instrument is an adaptation of the one designed by Terborg and Peters^18^ “Women as Managers Scale” (WAMS), the reason for its selection was its psychometric properties^16^ and the smaller size, which prevented adding the sociodemographic questions to the questionnaire, it would be too long and tedious.

The questionnaire consists of 7 items, answered using a 7-point Likert scale, ranging from 1 “Total disagreement” to 7 “Total agreement”.

It presents two dimensions on the gender perspective:

1. The “Instrumental Characteristics” dimension: evaluates the extent to which people consider that women present characteristics commonly associated with leadership. 2. The “Acceptance of Female Leadership” dimension: which evaluates the level of acceptance of women as leaders.

Demographic and job-related questions were included to analyze their relationship with the acceptance of women leaders.

Therefore, the study variables are made up of three blocks:

1. Sociodemographic variables intended to identify the profile of the study population: age, sex, marital status, profession, and academic level.
2. Variables related to the job: years of work experience, years of experience in management/leadership tasks, position held, hospital classification according to number of beds, span of control, and number of categories in charge.
3. Result variables of the measurement instrument selected for the study, Acceptance of female leadership, Total scale, Instrumental Characteristics and Acceptance of Female Leadership.

### Analysis of data

Univariate descriptive analysis of the qualitative variables was carried out using frequency distribution measures and percentages of each category. To describe the quantitative variables, measures of central tendency (mean or median) will be given, as well as measures of dispersion (standard deviation).

In our census study, where the data obtained was from the entire population, it was not necessary to make statistical inferences to make accurate statements. Inferential statistical tests have been applied to evaluate the influence of sociodemographic and job variables on the study outcomes and determine if there were statistically significant differences or associations between these variables and those of the ACT-LM questionnaire. The relationship between the response variables and the quantitative variables was explored using an ANOVA analysis if the data followed a normal distribution, analyzed using the Shapiro Wilk normality test. The variables that did not comply were treated with the non-parametric Kruskal–Wallis test. To examine the relationship between the response variables and the qualitative variables, the Chi Square hypothesis test was used. In all cases the results were considered to reach statistical significance with *p* < 0.05.

### Ethics approval and consent to participate

The Clinical Research Ethics Committee of Aragon (CEICA) Favorable Report, file number PI21/009, and consent from the author of the ACT-ML questionnaire^14^ for its use, were requested and obtained. All methods were carried out in accordance with relevant guidelines and regulations. Informed consent was obtained from all subjects.

## Results

A total of 149 responses were received, resulting in a response rate of 71.98% (149/207). Five of them did not meet the inclusion criteria (primary care, head of medical service, privately managed hospital) and they were excluded from the analysis of the results, considering a total of 144 responses valid.

The results obtained from the analysis of the sociodemographic variables (Table 1) indicate that the leaders of the hospital physiotherapy units have an average age of 49.6 (SD 6.1), the majority are women (69.4%), they are married or living as a couple (77.1%) and they have diplomas (59.7%) in physiotherapy (91.0%).

**Table 1:** Sociodemographic variables of the participants.

Variable	n (%)
Sex	
Male	43 (29.9%)
Female	100 (69.4%)
Non binary	1 (0.7%)
Age	
Mean (Standard deviation)	49.6 (6.1)
Median	49
Minimum maximum	31/64
Civil status	
Single	21 (14.6%)
Married, in a relationship	111 (77.1%)
Divorced	11 (7.6%)
Widowed	1 (0.7%)
Profession	
Nurse	11 (7.6%)
Physiotherapist	131 (91.0%)
Speech therapist	1 (0.7%)
Occupational therapist	1 (0.7%)
Academic level	
Diploma	86 (59.7%)
Degree	26 (18.1%)
Master	26 (18.1%)
Doctor	6 (4.2%)

n: number of population subjects.

%: percentage of population.

The analysis of the variables related to the job position (Table 2) reveals that the majority of responders (77.8%) have more than 20 years of work experience, 43.8% have been performing management or leadership tasks for between 1 and 5 years, and it is worth noting that 34.8% have been there for between 6 and 15 years. It is also noted that the majority of those surveyed (47.2%) have experience in their current position ranging from one to five years.

**Table 2:** Variables related to the job position.

Variable	n (%)
Years of work experience	
< 1 year	0 (0.0%)
15 years	0 (0.0%)
6–10 years	3 (2.1%)
11–15 years	7 (4.9%)
16–20 years	22 (15.3%)
> 20 years	112 (77.8%)
Years of management/leadership experience	
< 1 year	12 (8.3%)
15 years	63 (43.8%)
6–10 years	27 (18.8%)
11–15 years	23 (16.0%)
16–20 years	8 (5.6%)
> 20 years	11 (7.6%)
Job that performs	
Coordination	19 (13.19%)
Unit supervisor	102 (70.83%)
Physiotherapy deputy director	8 (5.55%)
Area supervisor	14 (9.72%)
Head	1 (0.69%)
Hospital classification/No. of beds	
Cluster 1/< 200	33 (22.91%)
Cluster 2/200–500	59 (40.97%)
Cluster 3/501–1000	39 (27.08%)
Cluster 4/> 1000	13 (9.02%)
Span of control	
0–25 workers	56 (38.9%)
26–50 workers	56 (38.9%)
51–75 workers	14 (9.7%)
> 76 workers	18 (12.5%)
Number of professional categories under your responsibility	
1 category	6 (4.2%)
2 categories	27 (18.8%)
3 categories	33 (22.9%)
4 categories	43 (29.9%)
5 categories	23 (16.0%)
6 categories	9 (6.3%)
7 categories	1 (0.7%)
8 categories	2 (1.4%)

n: number of population subjects.

%: percentage of population.

Regarding the profile of the people who oversee a level 4 hospital, considered large hospitals with high management complexity, it is worth noting that only 46% are women, compared to 69.4% of female leader. Practically 78% of those surveyed have a maximum of 50 people and around four professional categories as a span of control.

The results obtained from the analysis of the outcome variables of the ACT-LM questionnaire (Table 3) are presented for each of the items in the questionnaire, as well as for the two dimensions and the total scale. It is observed that in all items, most responses fall into category “1” with the greatest acceptance of female leaders. Therefore, all average scores have values slightly above one.

**Table 3:** Result of the ACT-LM questionnaire.

Item	1	2	3	4	5	6	7	Mean	SD
Instrumental Characteristics								1.5	0.99
Women are not ambitious enough to be successful in the world of work	114 (79.2%)	6 (4.2%)	5 (3.5%)	8 (5.6%)	6 (4.20%)	4 (2.8%)	1 (0.7%)	1.63	1.38
Women are not competitive enough to succeed in the world of work	117 (81.3%)	8 (5.6%)	4 (2.8%)	2 (1.40%)	6 (4.2%)	3 (2.1%)	4 (2.8%)	1.59	1.46
Women do not have the necessary social and political skills for management positions	134 (93.1%)	2 (1.40%)	0 (0.0%)	2 (1.40%)	1 (0.7%)	1 (0.7%)	4 (2.80%)	1.28	1.16
Acceptance of female leadership								1.27	0.72
Women should have the same opportunities as men to participate in management training programs	140 (97.2%)	0 (0.0%)	1 (0.7%)	0 (0.0%)	0 (0.0%)	0 (0.0%)	3 (2.1%)	1.14	0.87
Women can rise to the same extent as men	119 (82.6%)	5 (3.50%)	8 (5.60%)	5 (3.50%)	4 (2.80%)	1 (0.7%)	2 (1.40%)	1.48	1.21
The work carried out by women leaders is just as valuable as that carried out by men leaders	134 (93.1%)	1 (0.7%)	4 (2.80%)	2 (1.40%)	0 (0.0%)	0 (0.0%)	3 (2.1%)	1.23	0.97
Women present the ability to acquire the necessary skills to become leaders	136 (94.4%)	2 (1.40%)	1 (0.7%)	1 (0.7%)	0 (0.0%)	0 (0.0%)	4 (2.80%)	1.22	1.03
Total scale								1.37	0.68

For each item: number of population subjects, (%) percentage of population.

*SD* standard deviation.

Considering that the best score on the scale would be “1”, we observe that the worst valued items (with the highest average value) are those that make up the Instrumental Characteristics dimension, with the respondents not fully agreeing that women are sufficiently competitive (18.7%) or ambitious (20.8%) to be successful in the world of work.

Regarding the dimension Acceptance of Female Leadership, the highest average value is given by 17.4% of respondents who do not totally agree that women can advance to the same extent as men.

After the analysis carried out to identify the factors that influence the results obtained in the ACT-LM Questionnaire, the results indicate that the demographic variables age, marital status, profession, and academic level have no statistically significant relationship with the results of the ACT-LM. On the contrary, the sex variable (Table 4) shows statistically significant differences between men and women in the Instrumental Characteristics dimension and in the total scale, being lower in the male sex, which indicates that men have greater consideration on female leadership capabilities than women themselves.

**Table 4:** Inferential statistics of the ACT-LM scale with the sex variable.

Characteristic	Subscales		Total scale
	Instrumental	Leadership acceptance	
Sex			
Male	1.14 (0.41)	1.20 (0.58)	1.18 (0.38)
Female	1.66 (1.13)	1.29 (0.78)	1.45 (0.76)
*p*	0.011*	0.072	0.027*

For each group: mean (standard deviation). Statistical significance *p* < 0.05.

Of the variables related to the job, the variable span of control and the variable number of categories in charge have a statistically significant relationship with the ACT-LM questionnaire (Table 5). In the control area, a statistically significant difference is seen in the instrumental subscale between the ranges between “51–75” on the one hand and “0–25” and “26–50” on the other hand, which, since they are not correlative, do not allow us to extract conclusions. In the variable number of professional categories in charge, the significant differences occur in the instrumental subscale between 3 categories, with 2, 4 and 6 or more; in the acceptance subscale between 3 with 4 and 5 and in the Total Acceptance of Female Leadership scale between 3 categories with 2, 4 and 5.

**Table 5:** Inferential statistics of the ACT-LM scale with variables related to the job position.

Characteristic	Subscales		Total scale
	Instrumental	Leadership acceptance	
Span of control			
0–25	1.67 (1.11)	1.28 (0.87)	1.45 (0.83)
26–50	1.51 (1.06)	1.24 (0.48)	1.35 (0.60)
51–75	1.00 (0.00)	1.29 (0.93)	1.16 (0.53)
≥ 76	1.31 (0.67)	1.31 (0.74)	1.31 (0.47)
*p*	0.049*	0.77	0.262
No. prof. categories in charge			
1	1.89 (1.57)	2.00 (2.45)	1.95 (2.06)
2	1.35 (0.92)	1.23 (0.46)	1.28 (0.51)
3	1.94 (1.19)	1.36 (0.58)	1.61 (0.69)
4	1.33 (0.89)	1.13 (0.55)	1.22 (0.47)
5	1.46 (0.89)	1.10 (0.22)	1.25 (0.43)
6 or more	1.11 (0.38)	1.50 (0.91)	1.33 (0.59)
*p*	0.010*	0.047*	0.034*

For each group: mean (standard deviation). Statistical significance *p* < 0.05.

## Discussion

The results of this research provide relevant information to understand the situation of female leadership in public hospitals in Spain.

The presence of 69.4% of female leaders of the physiotherapy units of public hospitals in Spain is a good result compared to the general statistics of female physiotherapists in Spain which, according to the report of the General Council of Colleges of Physiotherapists^19^, was 62.4%. This is encouraging and aligns with the European Parliament Resolution of 14 March 2017 aimed, among other things, at reducing the under-representation of women in management positions^9^. Unfortunately, this high percentage is not reproduced when we talk about highly complex hospitals, in which the presence of women in charge of their management is reduced to 46%. This data would coincide with the findings of Rincón^4^, Saadoun^20^ and LaPierre and Zimmerman^21^ who reported that women are less likely than their male colleagues to aspire to high-level leadership positions.

Regarding work experience, 77.8% of the population studied has more than 20 years of experience, which indicates extensive training in clinical physiotherapy. This result coincides with the statement of physiotherapy leadership experts who state that clinical experience is essential to be successful in the leadership of physiotherapy units^22^.

The position held most frequently is that of unit supervisor, with 70.83%, this corresponds to the organization of most hospitals, in which the physiotherapy units depend on the nursing directorates. Special mention deserves the 8 (5.55%) physiotherapy deputy director of the Valencian region, their management is independent of the nursing, depending directly on the hospital management. Furthermore, there are no gender differences in access to this position.

It is relevant to note that physiotherapists present similarities between the years they have been performing their current position and the years performing management and/or leadership tasks, which indicates that they do not hold other management positions, evidencing that there is no mobility in this professional area. In professions such as nursing, it is common to go through different positions and levels of leadership. This fact is considered an obvious barrier to the professional development of physiotherapists, both men and women, in the field of leadership^22^, since they have practically no possibility of accessing senior management positions.

The analysis of the acceptance of female leadership in the context of hospital physiotherapy units revealed a significant improvement with respect to the results obtained by the author of the ACT-LM questionnaire^16^, especially in men’s assessment of female leadership, since it obtained means of 2.34 (SD 1.15) in men and 1.92 (SD 0.94) in women. Our results are more consistent with current social trends of equality, since the vast majority of respondents showed high acceptance of women leaders, reflecting a positive evolution in the perception of women's leadership capabilities^4^.

The items that make up the Instrumental Characteristics dimension showed worse results than those of the Acceptance of Female Leadership dimension. This indicates that, although leaders of physiotherapy units mostly accept female leadership^23^, they also reflect the belief that women may lack, to some extent, some characteristics that are considered necessary for effective leadership.

It is relevant to note that the women surveyed themselves showed a lower rating in both the two dimensions and in the total scale, a difference that was significant in the Instrumental Characteristics dimension. This perception could be due to personal barriers, since some studies^15^ indicate that women may have lower self-esteem and feel less prepared to hold leadership positions compared to men.

The concept of ambition also emerged as a point of reflection. The standards of motivation, high competitiveness and ambition necessary to achieve success can contrast with the idealization of motherhood and the roles traditionally assigned to women^24^. It is important to consider how these gender stereotypes^4^ can influence the perception of women leaders and their professional self-assessment.

In general, physiotherapists who occupy leadership positions in public hospitals in Spain show a high level of acceptance of female leadership, but there are still beliefs that can improve regarding the acceptance of female leaders, considering that women have the same leadership characteristics than men and who have the same possibilities of promotion, are those with the greatest room for improvement. Promoting gender diversity in leadership is beneficial for society as a whole^4^, since it has been shown that women tend to act from ethical values, such as benevolence and universalism^25^.

Despite the progressive progress that has occurred in the presence of women in leadership positions, we still find room to continue researching and working in this direction and thus eliminate all negative gender stereotypes about leadership and women.

This study highlights the importance of recognizing and challenging gender stereotypes, which persist today, to achieve gender equality in the field of physiotherapy leadership. Allowing people to express and develop without limitations based on their gender will contribute to greater equity and diversity in leadership positions and will ultimately benefit patients, the profession, the healthcare system and society as a whole.

A limitation of this study is that the autonomy in health policy of the regions does not allow us to know exactly the competencies of each of the leadership positions that appear in this study.

## Conclusions

In conclusion, most of the leaders of the physiotherapy units in public hospitals in Spain are women, although this is reversed in favor of men in highly complex hospitals. There is still a stereotype that women do not have enough ambition and competitiveness to succeed in the world of work.

## Data Availability

The raw data supporting the conclusions of this article will be made available by the corresponding author, without undue reservation.
